# The high-incidence period of *Mycoplasma pneumoniae* infections 2023/2024 in southeast Germany was associated with a low level of macrolide resistance

**DOI:** 10.1007/s15010-024-02336-4

**Published:** 2024-07-01

**Authors:** Roger Dumke

**Affiliations:** Institute of Medical Microbiology and Virology, University Hospital Carl Gustav Carus, Technische Universität Dresden, Dresden, Germany

**Keywords:** Community-acquired pneumonia, *Mycoplasma pneumoniae*, Macrolide resistance, 23S rRNA

To the Editor,

*Mycoplasma pneumoniae* is a member of the cell wall-less *Mollicutes* group and a common cause of respiratory tract infections in humans. Despite the mild and self-limiting course of many cases, severe manifestations with symptoms of interstitial pneumonia and extra-pulmonary complications might occur. In high-incidence periods, up to 40% of cases of community-acquired pneumonia can be attributed to *M. pneumoniae*. Epidemiology shows a typical pattern with an increase in infections every 3 to 7 years [1]. Due to the intrinsic resistance of these bacteria to betalactam antibiotics, treatment of infections is challenging. Alternative therapeutic options (tetracyclines and quinolones) have side effects among the most affected population group of children. Thus, macrolides are the first-line antibiotics. Unfortunately, consistently high rates of macrolide resistance up to 100% of strains have been described especially in Asia [2]. In contrast, rates in many countries in Europe remain below 10% in recent years [3]. Resistance to macrolides is based on different single mutations (A2063G/C, A2064G/C and C2617G/A) in the peptidyltransferase loop of domain V of the 23S rRNA of *M. pneumoniae* resulting in a high-level resistance > 16 µg/ml to azithromycin.

During the SARS-CoV-2 pandemic with corresponding measurements taken to reduce the transmission of respiratory pathogens, *M. pneumoniae* infections decreased strongly [4]. Meanwhile, re-emergence of *M. pneumoniae* infections during 2023/2024 has been reported in different areas worldwide [5]. Accurate detection of cases is hampered by a missing obligation to report these infections in many countries. In Germany, reporting is mandatory in only one federal state (Saxony). Figure 1 shows the number of infections (confirmed by serological and molecular methods) since 2014, demonstrating a strong increase in cases in the 2023/2024 season. Especially in the year 2024, the incidence was greater than that in all other pre-pandemic years considered.

**Fig. 1:**
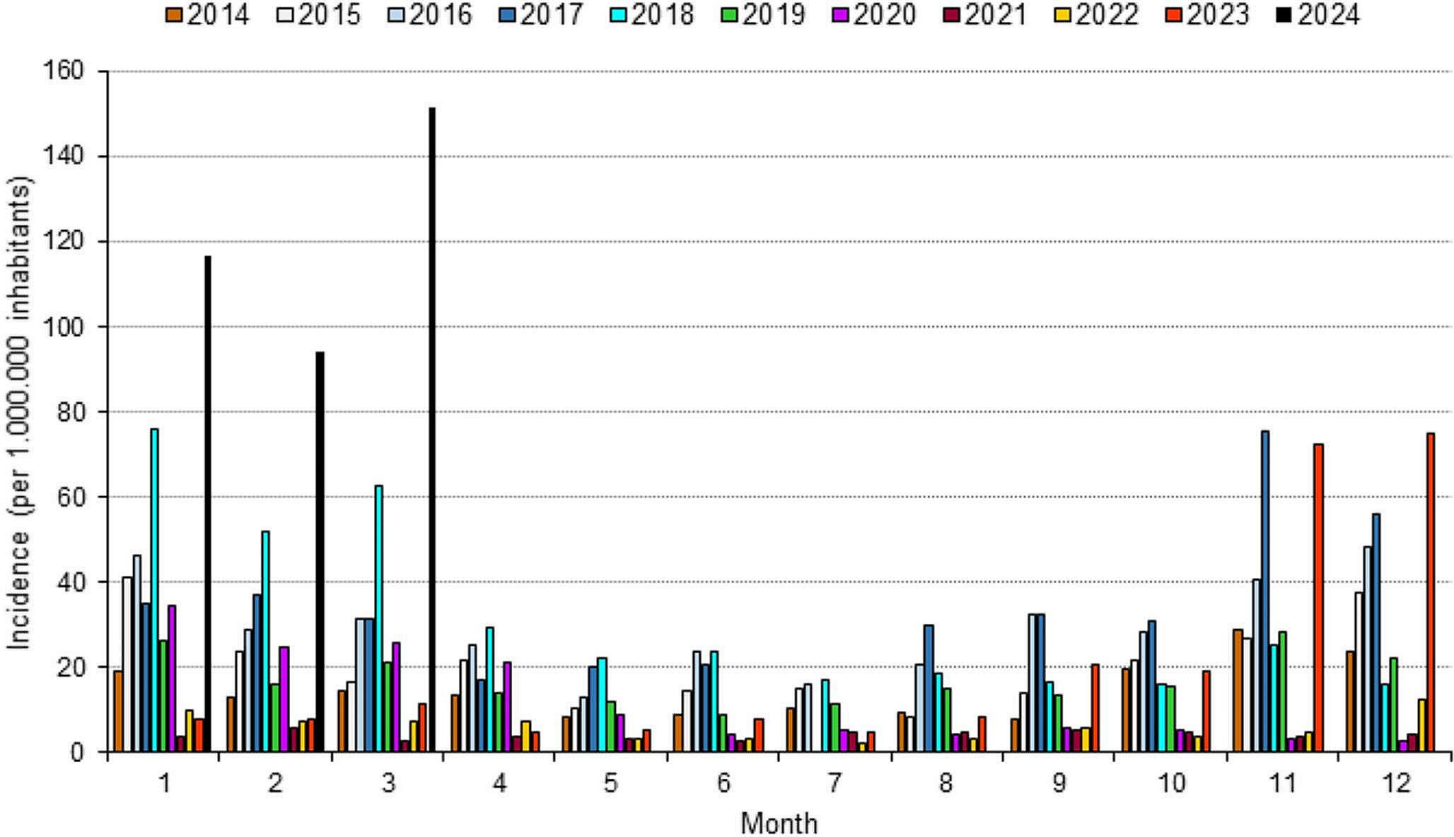
Incidence of reported *Mycoplasma pneumoniae* infections in the federal state Saxony, Germany, January 2014-March 2024 (source of number of cases: https://www.lua.sachsen.de)

To characterize the rate of macrolide-resistant strains during the period of increased incidence of infections in southeast Germany, clinical materials (nasopharyngeal swabs) from *M. pneumoniae*-positive individuals were tested by different real-time PCR approaches in four laboratories (2xBerlin, Dresden, Leipzig) investigating samples from general and pediatric practices in the surrounding catchments. Between November 2023 and March 2024, these laboratories stored (-20 °C) all *M. pneumoniae*-positive samples. Second samples from the same patient were excluded. An 823 bp fragment (nt 1,822 to 2,644) of the 23S rRNA of *M. pneumoniae* was amplified and Sanger sequenced. The sequencing results were compared with the corresponding part of the *M. pneumoniae* reference genome of strain NCTC10119 in GenBank.

Overall, 449 DNA samples were investigated and the amplification/sequencing results from 422 samples were evaluated. Based on the number of reported cases of *M. pneumoniae* infections between November 2023 and March 2024 in the federal state Saxony (*n* = 2,087), the approximate percentage of samples successfully investigated for macrolide resistance accounts for 14%. In 11 strains (2.61%), a mutation in the 23S rRNA of *M. pneumoniae* associated with macrolide resistance was confirmed (exclusively A2063G). We used the labor-intensive sequencing procedure to obtain information about mutations outside of the common 2063/2064 positions which are most frequently responsible for macrolide resistance. The rare 2617 transitions were not found in any of the samples. Additionally, the C2353T mutation recently described in strains in Vietnam [6] could not be detected. In two samples, an A2228T or G2395A mutation of 23S rRNA was confirmed. However, their importance for macrolide susceptibility is unknown.

To our knowledge, this is one of the first reports quantifying the macrolide resistance among *M. pneumoniae* strains in an European country during the recent epidemic period. Due to the comparable epidemiology of *M. pneumoniae* infections in the season 2023/2024, the cross-border exchange of people and the similar regimes of macrolide prescription in central Europe, it can be assumed that changes of rates of macrolide resistance in other countries of this region are limited [7]. However, further studies have to confirm this hypothesis. The rate of 2.61% among strains circulating 2023/2024 in southeast Germany is in agreement with previous investigations in Germany (2009–2012: 3.3%, 2016–2018: 3.0%). Consequently, macrolides remain the antibiotics of choice in cases in which a treatment of infection is necessary. Nevertheless, therapy failure is an indication to test the macrolide susceptibility of a strain in a specialized laboratory. Furthermore, it can be expected that the resistance pattern of circulating strains had only a small impact on the number of infections during the season of high incidence this year in Germany and other reasons (lifting of nonpharmaceutical interventions against COVID-19, emergence of differing genotypes in comparison to the years before, decreased herd immunity) are responsible for the increased number of *M. pneumoniae* infections.

## Data Availability

No datasets were generated or analysed during the current study.
